# Initial programme theory development: The first step in a realist evaluation of a cross-sectoral intervention for expectant Danish parents living with psychosocial risks

**DOI:** 10.1371/journal.pone.0295378

**Published:** 2023-12-20

**Authors:** Sara Mandahl Ellehave, Louise Lund Holm Thomsen, Marianne Stistrup Frederiksen, Charlotte Overgaard

**Affiliations:** 1 Public Health and Epidemiology Group, Department of Health Science and Technology, Aalborg University, Aalborg East, Denmark; 2 Clinical Nursing Research Unit, Aalborg University Hospital, Aalborg, Denmark; 3 The Unit of Health Promotion, Department of Public Health, University of Southern Denmark, Esbjerg, Denmark; National Institute of Public Health: Instituto Nacional de Salud Publica, MEXICO

## Abstract

A distinct inequality in maternity care exists, and women with psychosocial risks are at a greater risk of adverse birth outcomes. In several high-income countries, a psychosocial risk assessment early in pregnancy is recommended so that expectant parents are offered an appropriate level of care which facilitates relevant, tailored interventions for those in need. In 2017, a cross-sectoral and interdisciplinary intervention for expectant parents with psychosocial risks was developed and implemented in the North Denmark Region. The development process of the intervention has not been reported and theory-based knowledge about how supportive interventions bring about change for expectant parents with psychosocial risks is scarce. Through the initial phase of a realist evaluation, we aimed to elicit key contexts and mechanisms of change regarding the intervention for expectant parents with psychosocial risks. Through an initial programme theory, this article illustrates how, for whom and in which contexts the intervention is intended to work. Data is comprised of intervention documents, 14 pilot observations and 29 realist interviews with key stakeholders. A thematic analytical approach inspired by retroductive thinking was applied to identify and analyse patterns related to the incentive of the intervention, its structure, intended outcomes, generative mechanisms and contextual matters. Generative mechanisms responsible for bringing about change in the intervention were identified as healthcare professionals’ approach, continuity, trust, early intervention and social network. Cross-sectoral collaboration and healthcare professionals’ competencies were assumed to be central stimulating contextual factors. The initial programme theory developed in this study will serve as the basis for further refinement via empirical testing in a later phase of the realist evaluation.

## Introduction

Maternal and child health is characterised by significant and consistent social inequality [1, 2]. Women with psychosocial risks due to, e.g., low educational level, poor living conditions, poverty, psychological distress, social isolation, substance abuse or exposure to violence or abuse are at an increased risk of maternal morbidity and adverse birth outcomes such as preterm birth, stillbirth, low birth weight and perinatal mortality [3–5]. Their children’s long-term health and life chances are also negatively affected [6, 7]. These women and their partners’ needs are often complex and antenatal services should therefore be individualised and tailored [8, 9].

In several high-income countries, national guidelines recommend a psychosocial risk assessment early in pregnancy in order to assign expectant parents an appropriate level of care and offer relevant, tailored interventions [10–12]. The methods used for psychosocial risk assessment have however been questioned [13, 14]. Specifically, the use of checklists or close-ended questions may not allow expectant parents to share sensitive and potentially retraumatising issues thus preventing disclosure of important information or engagement with services [14]. Psychosocial risk assessment may furthermore trigger negative feelings of, e.g., fear, stigma or loss of self-esteem among expectant parents [15–18]. Awareness of such potential negative consequences of public health interventions is important to ensure that expectant parents receive quality care that meets their support needs. Interventions or programmes for expectant parents with psychosocial risks are rarely the focus of in-depth studies and often lack formal evaluation [9, 19, 20].

In Denmark, maternity care is provided at four levels according to the nature and severity of parents’ psychosocial risk factors [11]. Following Danish national guidelines [11], the Regional Health Authority and the municipalities of the North Denmark Region developed and implemented a cross-sectoral and interdisciplinary intervention for expectant parents with psychosocial risks. This intervention, as assessed in this study, was delivered by midwives (regional sector) and health visitors (municipal sector) in 2017 [21] and is still currently used in the region. The target group for the intervention was expectant parents with complex mental health problems or social disadvantages of either an economic or social character [11, 21]. The intervention was organised into three sequential activities. First, all expectant women and their partners (if attending) would undergo a systematic psychosocial risk assessment by use of a dialogue-based, semi structured interview guide at the first antenatal midwife consultation in order to identify psychosocial risk factors as well as protective factors and resources in the family. If risk factors were identified, the expectant parents were invited for a cross-sectoral team meeting involving the parents, their health visitor and their midwife with the aim of planning a targeted and tailored support plan. Supportive services were then offered, ranging from high-frequency consultations with the health visitor or midwife to cross-sectoral and interprofessional services such as courses specifically tailored for parents with psychosocial risks and offered in small groups and with continuity of course leaders. Overall, the intervention was designed to improve the parenting and coping skills of the expectant parents and reduce adverse birth outcomes and perinatal mental health problems by, e.g., reducing parental stress levels [21].

The intervention was based on the notions of a local intervention reported to show promising results [22]. It was adapted to the wider context of the North Denmark Region and upscaled to include all three maternity units and 11 municipalities in the region. Essential components were changed during the upscaling process. The adaption from a local to a regional setting was not preceded by scientific evaluation, nor were pilot or feasibility studies undertaken [21]. The intervention components have been described in a brief report targeting health professionals [21] and in lay information material, but the development process of the intervention has not been reported in writing. Potentially unintended and negative consequences of the psychosocial risk assessment and service tailoring for expectant parents with psychosocial risks have been highlighted in recent studies [15, 17, 23]. These uncertainties challenge the adaptive and scalable potential [24] of the intervention and therefore warrant scientific evaluation. The aim of our study was to elicit an initial programme theory as the first phase of a realist evaluation of the intervention for expectant parents with psychosocial risks in the North Denmark Region.

## Methods

### General study design

We conducted the initial phase of a realist evaluation using a qualitative approach. The realist methodology draws on critical realism and scientific realism, respectively, [25, 26] and provides a generative understanding of the causality of complex interventions through which explaining how and why an intervention works is possible [27, 28]. Critical realism projects the understanding of a stratified mind-independent reality, divided into three related domains—the real, actual and empirical–and focuses on exploring, via retroduction and abduction [29], underlying, mind independent structures and mechanisms (the real domain) that when activated by certain stimulating contexts will generate outcomes (the actual domain) that potentially become observed (the empirical domain) [28, 30]. Critical realism hence contributes a vertical, ontologically deep and generative understanding of causality rather than the more linear, simple explanation of causal relations [31]. Scientific realism shares the understanding of a mind independent reality being accessed via retroduction [32] and further contributes to realistic evaluation through the notions of the nature of reality being captured by scientific theories and of the best, most credible theories approximating the truth [26, 32]. The purpose of realist revaluation is thus to generate ontologically deep scientific explanations about how a programme works by unpacking the generative mechanisms and their conducive contexts responsible for outcomes of interest as well as unintended outcomes of a programme [32]. More specifically, a realist evaluation goes a step further than standard causal explanations by allowing us to understand how the effects of a programme are produced by opening the “black box” and via constructs of context-mechanism-outcome configurations (CMOc) illustrating what (and how) underlying mechanisms and structures affect the process between input and outcome [33]. A context is defined as the external individual, organisational or environmental circumstance or condition in which the programme is implemented [28]. Mechanisms are defined as the resources provided by a programmes’ modalities and the way people respond to those resources [28, 32, 34], while outcomes are defined as the intended and unintended effects generated by certain combinations of contexts and mechanisms [28]. CMO configurations make the substance of the programme theory in realist evaluations [28], which is an essential prerequisite of realist evaluation [28]. The overarching theory or model of how an intervention is expected to work is described as a programme’s rationale and assumptions about contexts and mechanisms that connect the programme’s inputs to outcomes–both intended and unintended [35]. The ‘theory’ in a programme theory “may be an articulation of practice wisdom or of tacit assumptions–that is, not only a formal, research-based theory” [33, p. 33]. The programme theory we developed drew on Funnel & Rogers’ elaboration of how to present programme theories [36]. Intended and unintended effects of the intervention may also become apparent through in-depth theorisation of a programme theory [28, 37]. Knowledge about both the intentional and unintentional workings of an intervention is a considerable advantage of adopting the realist approach. This is partly because the contribution will ensure that the benefits of the intervention outweigh its harms and partly because a detailed understanding of generative mechanisms may facilitate refinement and improvement of the intervention and thus enable upscaling and replication processes [28]. Following the understanding of realist evaluation, i.e., that people are not passive recipients of interventions and that a programmes’ ability to bring about change is highly dependent on its participants’ commitment, [28] adopting a realist perspective on evaluation of the intervention for expectant parents with psychosocial risks also enhanced our attention regarding social interaction between the expectant parents and healthcare professionals (HPs).

### Data collection

**Fig 1:**
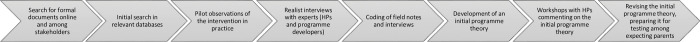
Process of the gleaning phase: development of the initial programme theory.

Following the realist evaluation cycle, the first step represents the theory gleaning phase, also referred to as theory elicitation [28, 38] (Fig 1). During this phase, we developed an initial programme theory which captured the assumptions of key stakeholders and programme developers regarding how the intervention for expectant parents with psychosocial risks is intended to work. To obtain and articulate a preliminary understanding of how, why and under what circumstances the intervention works, we adopted an elucidating research approach which allowed for a row of data collection methods and sources of information [39]. First, formal documents describing the intervention were collected from key stakeholders. These documents, included the written reciprocal health agreement framing the intervention, an implementation guideline for the intervention and the interview guide used for the systematic dialogue based psychosocial risk assessment [21]. Additionally, the first author conducted supplementary searches of relevant databases (Google Scholar, PubMed, Scopus, Sociological Abstracts) and web pages (Australian Government–Department of Health and Aged Care, National Institute for Health and Care Excellence, and Danish Health Authorities [40–42]) using the search terms “intervention”, “programme”, “guideline”, “antenatal”, “perinatal”, “pregnant” and “psychosocial risks”. These searches were made to identify similar interventions worldwide and international evidence or guidelines regarding support for expectant parents with psychosocial risks and helped us identify key policy reports and relevant research articles on the topic.

To further enhance and articulate our preliminary understanding of relevant contexts and mechanisms triggering the outcomes of the intervention, pilot observations [43] of the delivery of key modalities of the intervention were conducted. Two authors (SME & LLHT) observed midwifery consultations with psychosocial risk assessment of expectant parents and team meetings. These observations were made with the overall purpose of the researchers learning about the field and allowed us to gain insight into the delivery of the intervention and the dynamics between expectant parents and midwives and/or health visitors. Twenty-nine *realist interviews* were then conducted [38, 44, 45]. The interviews were, like the observations, conducted by two authors (LLHT & SME) both of whom were experienced in qualitative research methodology. The interview guide was developed with the purpose of strengthening and elaborating the initial CMOcs elicited from the intervention documents and research articles. The interview guide started off with exploratory questions with attention being given to particular context as the intervention was implemented across multiple settings. This was followed by more targeted questions which explored the impact of contextual factors and mechanisms through questions such as *“Please try to explain to me what you think it is in the aforementioned activity that makes the pregnant woman benefit from it*?*”* and “*In your experience*, *what circumstance are in place for this activity to be successful/when this activity is not successful*?” Finally, questions about potentially unintended consequences of the intervention were asked, e.g., *“Are there any situations you can think of where this (activity) does not lead to the intended effect*?*”*. The interviews lasted between 32–57 minutes.

Parallel to the realist interviews, SME and LLHT observed 14 antenatal midwife consultations and team meetings that aimed at strengthening the refinement of elicited contexts and generative mechanisms important to the intervention and eliciting potential new ones. The authors were focused on both verbal and physical behaviours. Questions like *“How is the midwife’s phrasing concerning the assessment for psychosocial risk factors*?*”*,*”How does the pregnant woman respond (verbally and non-verbally)*?*”* and “*What is the role division between health visitor and the midwife during the team meetings*?” served to focus our observations which were documented as field notes [43, 46].

Realist interviews and observations were conducted over an eight-month period (from November 2020 to June 2021) with the aim of gaining a deep insight into the stakeholders’ assumptions regarding the workings of the intervention. Due to COVID-19 restrictions, not all midwife consultations allowed for observations due to small consultation rooms. Most informants were interviewed via Microsoft Teams or by telephone. Nonetheless, some face-to-face interviews were completed.

For the final part of the theory gleaning phase of the evaluation and to decide on in- and exclusion of content in the initial programme theory, frontline workers and other key stakeholders were invited for workshops where researchers and programme providers met face-to-face to comment, discuss, adjust and refine the initial programme theory.

### Sampling and recruitment

Programmes are often developed and implemented within organisations, and members of an organisation are thus essential for explaining the anticipated impact of a programme [36, 47]. The intervention was implemented in the entire North Denmark Region and comprised of three maternity units, midwifery out-patient clinics in all larger towns in the region and 11 municipal health visiting units. Eight of the 11 regional municipalities agreed to participate in the initial phase of the evaluation. We strove to achieve maximum variation in the data to accommodate expected implementation differences. Using a purposive sampling strategy [48], informants were recruited for interviews because of how their position within healthcare related to the intervention. Considering that this was the initial phase, informants represented expert level of experience with the intervention and were key representatives from the regional reference group. These experts included the regional project manager, chief midwives from each of the three maternity units, managers of the involved municipal health visiting units, a representative of general practitioners and a regional outpatient clinic for families with psychosocial risks. Informants primarily represented the decision level to secure insight into the incentives for and the development and implementation of the intervention [28, 36]. Key stakeholders from the decision level helped identify other relevant stakeholders who were actively engaged in the intervention through their daily practice; hence, more organisational levels were represented. The informants were considered to hold similar professional characteristics, emphasising a sample with high specificity [49]. Informants holding characteristics that were highly specific for the study aim underpinned the information power [49]. Twenty-nine informants were recruited for participation in the realist interviews (Table 1). Expectant parents will be included in a subsequent sub study with the aim of testing some of the initial programme theories derived in this current study.

**Table 1:** Characteristics of data collecting activities and informants contributing to the initial programme theory.

Data collection activity	Informant ID	Professional characteristics of informants	Total number of informants
Regional level individual interviews	I_1	Administrative/regional level manager of the intervention	1
	I_15	Representative of general practitioners	1
	I_16	Obstetrician	1
	I_2, I_3, I, 11	Chief midwives	3
	I_5, I_9, I_17, I_18, I_19, I_20, I_23, I_24, I_25, I_26, I_27, I_28	Frontline midwives	12
Municipal level individual interviews	I_6, I_7, I_8, I_12, I_13, I_14	Health visitor managers	6
	I_4, I_10, I_20, I_21	Front-line health visitors	4
	I_29	Health visitor specialist	1
	**Pilot observation ID**	**Type of activity**	**Total number of activities**
Regional level pilot observations	PO_1, PO_3	Midwife consultations–psychosocial risk assessments	2
	PO_2, PO_4, PO_5, PO_6, PO_7, PO_8, PO_9, PO_10, PO_11, PO_12	Team meetings	12
	**Workshop ID**	**Professional characteristics of participants**	**Total number of participants**
Regional and municipal level workshop	W_2	Frontline midwives and health visitors, a health visitor manager, a chief midwife, and a vice chief midwife	19
Regional level workshops	W_1	Front-line midwives and a chief midwife	10
	W_3	Front-line midwives and a chief midwife	21
Municipal workshops	W_4	Front-line health visitors and a health visitor manager	14
	W_5	Front-line health visitors and a health visitor manager	6
	W_6	Front-line health visitors and a health visitor manager	6
	W_7	Front-line health visitors and a health visitor manager	13
	W_8	Front-line health visitors	5
	W_9	Front-line health visitors and a health visitor manager	9

### Data management and analysis

The different sources of data (Fig 1) played different roles in the process of uncovering generative causation. The formal documents describing the design and implementation strategy of the intervention was analysed mainly to identify themes attributed to the deliverers of the intervention, the intervention modalities and expected outcomes. The studies identified through the initial literature search were reviewed regarding the identification of CMOcs for development of the initial programme theory. As these studies mainly covered parents’ perspectives, the studies provided invaluable insights into the contexts and mechanisms potentially responsible for the workings of the intervention from the perspectives of expectant parents in particular.

The pseudonymised field notes obtained during the observations of both midwife consultations and team meetings were all read thoroughly several times by the first and second author (SME & LLHT) before being analysed using the thematic analysis approach [50]. These data provided important insights into the design and implementation of the intervention as well as the unspoken (not formally written) characteristics of contextual circumstances and generative mechanisms being responsible for the workings of the intervention. All interviews were pseudonymised in pursuance of EU legislation on data protection [51], transcribed verbatim and imported into the Qualitative Data Analysis Software NVivo 11 by one author (SME). We used a thematic analysis approach [50] to identify and analyse data patterns in the interview transcripts. The analysis was performed using researcher triangulation. Two researchers (SME & LLHT) individually coded the transcripts following a coding manual. Initial codes were generated abductively based on the realist understanding of elements in a programme theory [28, 52, 53]. Data excerpts related to the theoretical background of the intervention, activities embedded in it, possible mechanisms, intended outcomes and influential contexts were coded. Any disagreements or ambiguities related to how to code data were discussed by the team. The thematic analysis was an iterative process during which we checked if the codes worked in relation to the entire data set and captured new emerging mechanisms. Coding occurred when an observable CMOc or parts of it was found in the data and memos were added in NVivo for review/refinement. Thus, the analysis evolved inductively when new patterns added to contexts, mechanisms or outcomes as they appeared from the data. The identified themes formed the foundation for formulating hypothesised CMOcs using retroduction. Within the realist tradition, retroduction includes investigating the underlying generative mechanisms leading to potential outcomes even though these mechanisms may not be directly observable empirically [27]. Retroduction is an empirical process of developing a theory which requires moving transcendentally from information collected by a researcher regarding a concrete phenomenon to the reconstruction of the basics for a deeper causal understanding [29, 54]. We therefore used our spontaneous interpretations [29] of information gathered through the formal documents of the intervention, other research on the topic, our field observations and interviews to generate our hypotheses of the generative causation of the intervention for expectant parents with psychosocial risks. We did this by attentively exploring how certain outcomes were expected to arise by both stakeholders and developers. We also examined what it was about the midwives’ and health visitors’ actions and practices they assumed would matter for reaching intended outcomes [32]. To assist this retroductive inquiry, we aimed to identify the mechanisms responsible for the effects of the intervention in certain contextual settings as inspired by Dalkin et al.’s (2015) elaboration detailing how resources and reasonings are mutually constitutive of a mechanism. We therefore disaggregated the mechanism into resources and reasonings whereby mechanisms could be identified by looking for statements indicating how a resource of the intervention introduced among the expectant parents with psychosocial risks was expected to trigger a change in their reasoning eventually forming the outcome. We looked for patterns in the data and were thereby able to connect resources and reasonings to outcomes that were related to certain contextual factors and were thus able to develop the CMOcs of the intervention. Any CMOcs resembling already identified CMOCcs were combined and, if appropriate, further refinement occurred.

The reporting of the study followed the COREQ consolidated criteria for reporting qualitative research [55] and reporting standards for realist evaluations [56].

### Ethical considerations

Under Danish legislation, qualitative studies are based solely on informed participant consent and are not subject to approval by a national health research ethics committee [57]. This study was complied with the General Data Protection Regulations of the European Parliament and the Council of The European Union [51]. In accordance with the principles outlined in the Helsinki Declaration [58], all informants were informed about the purpose of the study, how their data would be protected and that they had the opportunity to withdraw from the study at any time for any reason. Subsequently, all informants gave their written informed consent to participate [51].

## Results

**Fig 2:**
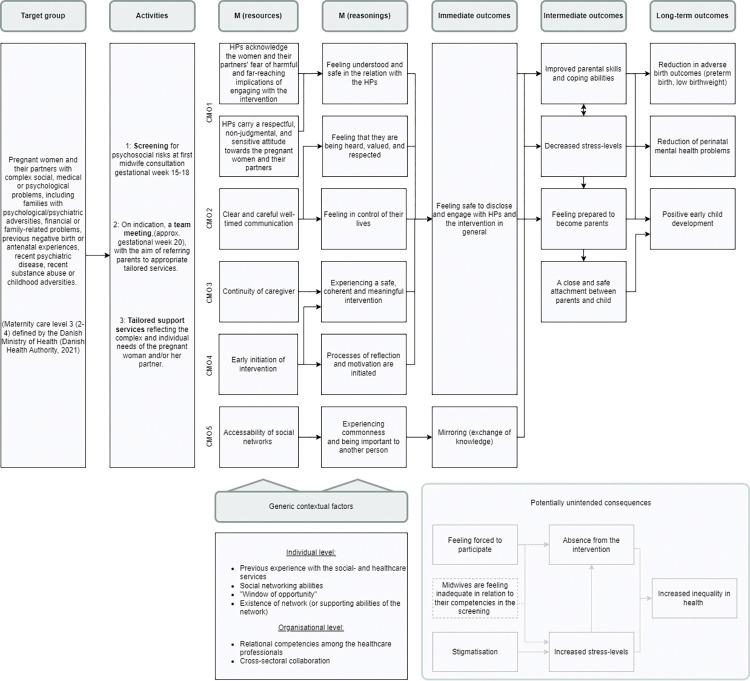
Initial programme theory of the cross-sectoral intervention for expectant parents living with psychosocial risks in the North Denmark Region. Elements relating to the organisational part of the programme theory are marked with dotted lines. Potential unintended consequences are marked with the lighter coloured lines.

Five dominant CMOcs suggesting different pathways through which expectant parents with psychosocial risks and their partners were expected to achieve the intended outcomes as well as potential unintended consequences were derived. They are visualised in the programme theory presented in Fig 2.

### CMOc 1 –Acknowledgement and respect

Previous experiences with the social services (C) were identified as a generic contextual factor with the potential of affecting the woman and her partner’s inclination to engage with HPs during the psychosocial risk assessment and team meetings (O) as explained by a health visitor manager:

They live on the experiences they carry: If dad has been in a marriage previously where the child was removed, then of course there is resistance (I_8).

HPs were aware that engaging with the intervention was not necessarily easy for parents due to their past experiences and that there was an inherent power imbalance in the relationship with the HPs:

The parents are well aware that this is about them becoming parents and that is where the fear comes in[…] We can work with their fear, but I don’t think we can eliminate it. I don’t think we are able to remove the premise and the imbalance or structure of power that lies within this assessment (the psychosocial risks assessment), but we can work with their fear and communicate openly about the purpose of the assessment. Namely, we give the parents the best conditions for pregnancy and parenthood (I_2).

Due to parents’ past experiences and the potential fear they may have felt, HPs’ knew that their approach towards the parents was important to make them feel safe and secure. HPs’ showing acknowledgement and belief in the couple as capable parents (M resource) and supporting their confidence was assumed to sustain self-belief and subsequently sense of parental skills (O). A midwife explained how she found a respectful approach crucial when she was asked about the generative mechanism for a trustful relationship between herself and the expectant parents:

When the screening and dialogue work well, she (the pregnant woman) will leave the consultation with a sense of trust of the system, trust in the midwife, and a feeling of empowerment in relation to what she has talked to the midwife about. She will be confident in the midwife being there for her regarding what she brought up at the consultation […] (And she may) experience that the midwife has helped her by pointing out precisely what her situation is, which is important to recognize when having a child and to her position in general. The mother may feel that she is not on her own while dealing with her challenges. She may feel that the midwife wishes her well and that she [the midwife] can help her receive support from the system, e.g., gain a social network, speak to the right healthcare workers, and receive the support she needs (I_3).

This excerpt illustrates the assumption that a respectful, non-judgmental and empowering attitude on the part of the HPs (M resource) may allow the expectant parents to feel understood and safe enough in their relationship with the HPs (M reasoning) to open up, share sensitive information and discuss support needs (O). Another excerpt showed how sensitive building the initial trust can be:

When I get close to what is sensitive, I am rejected. I point out the vulnerabilities and then it is not me they want to visit the next time […] When I get close to the heart, I have experienced [being rejected] a few times since we started screening the families. It may be because I am too crude, too old …or because I dare to ask these questions. That is a little difficult, actually (I_27)

Overall, acknowledgement (M resource) and sensitive articulation of the expectant parents‘ potential fear of being labelled as inadequate or being reported to the social services and eventually having their child removed was described as essential for the parents to feel safe in the relation to the HP (M reasoning) and was assumed a necessity for them to engage with the HPs and for the effect of the intervention.

### CMOc 2 –Clear and careful communication

Having a history with social services (C) and experiencing uncertainty or even fear in the presence of authorities was assumed to sometimes lead expectant parents to feel a loss of control or feelings of unworthiness as parents. Clear and careful communication during psychosocial assessment and team meetings (M resource) was seen to contribute to the expectant parents’ feelings of being heard, valued and respected as well as their ability to feel as if they were in control of their lives (M reasoning) in general. Feeling in control, valued and respected was assumed vital for expectant parents having a voice which pointed to their perpetual right to choose and framed engagement with the intervention as safe and harmless (O). Accordingly, it was considered essential to build trust for the expectant parents by HPs being transparent about the purpose of the meeting:

When we talk about what it is… why are we here. […] It is about articulating why we are doing the things we are, so that there is no hidden agenda. […] It is all about being open about the things we are doing

so that the expectant parents have no doubt about our position… They will have no reason to think that I am doing anything behind their back… […] This gives a trusting collaboration (I_5).

And:

It is our experience that we will not be able to build a trustful relationship with the couples until we are able to help the pregnant woman or her partner comprehend what we are doing, why we are doing it and what they may get out of it (I_3).

These citations underpin the assumption that HPs who speak openly and are informative (M resource) may sustain a feeling of respect and eventually trust in their relationship with the expectant parents (M reasoning). A midwife explained how clear communication and the timing of the risk assessment during the first consultation was important for her to accommodate expectant parents:

Telling them the purpose is one of the most important things I do to make them feel safe enough to share with me […] I also put an effort into establishing a relation before conducting the psychosocial risk assessment. When I was a student and introduced to the assessment, I sat with a midwife who did it for the first time, and I had to learn to do it [myself]. Back then, we did it as the first thing when they arrived. I do not do that any longer because I think building the relationship is crucial for me to be allowed to ask these questions and allow them to build trust in me. Therefore, I always place it last (I_26).

The midwives expressed how they frequently felt uncomfortable and inadequate when assessing the expectant parents for psychosocial risk factors. These experiences were generally shared by the midwives, and many referred to the interview guide as overwhelming. A midwife at the workshop expanded this perspective in the following manner:

That is how I experience it, too; it gets better the more you practice. In the beginning, I asked my colleagues and sought professional feedback quite a bit because I found that it was uncomfortable. I thought it was a strenuous task. I did not think it was my responsibility at all (Midwife during workshop W_1).

The data showed how specially trained HPs with qualified communicative skills (C) represent important moderating contextual factors to ensure well-timed and informative communication (M resource) assumed to be essential in making expectant parents feel safe (M reasoning) and share their stories (O). For the midwives to be able to ensure well-timed and informative communication, and covering all the themes in the interview guide, having enough time for the psychosocial risk assessment was pointed out as extremely important. Time available (C) was thereby identified as another contextual factor affecting the intervention.

### CMOc 3 –Continuity of caregiver

In the target group, many had multiple interactions with professionals and various authorities in relation to childbearing and other aspects of their lives. In some cases, these contacts led to distrust. Some women or their partners suffered from anxiety, social phobia, a history of loss or neglect which led to them being challenged regarding their ability to build relationships and trust in general. Negative experiences with professionals (C) together with challenged social networking abilities (C) were considered contextual factors that may affect expectant parents’ inclination to engage with HPs (O). Care by known HPs (M resource) was described as an essential generative mechanism that strengthened expectant parents’ inclination to reach out and share their experiences (O), as familiarity and predictability were assumed essential for expectant parents to feel comfortable and safe (M reasoning). This made these characteristics a prerequisite for building trust with the HPs (O). How a sense of security and familiarity with HPs was perceived by HPs as important for the woman and her partner is illustrated by the following excerpt:

A sense of security is linked to the fact that they know us. You will have the courage to join the café though you are challenged and do not attend frequently (I_5).

Knowing the HP may affect expectant parents with psychosocial risks and encourage them to feel safe and attend services which may otherwise trigger a sense of insecurity in these parents. One health visitor manager explained how they try to avoid change in caregiver to accommodate for continuity:

We believe in the value of the professional relation and the security that lies within this relation. Therefore, we strive to work in a way that allows us to avoid shifts in the professional relations if possible. That is a sustaining principle to us (I_7).

Continuity of HP was described as better facilitating tailored and more individualised services, as continuity ensured a well-prepared HP held in-depth insights into the family’s challenges and support needs. Continuity of HP (M resource) was thus considered a key mechanism for expectant parents to experience the intervention as meaningful (M reasoning). Not having to repeat one’s story to shifting HPs was additionally described as triggering feelings of meaningfulness, comfort and trust on the part of the expectant parents (M reasoning). This assumption was supported by an observation of a team meeting (PO_4) with a woman with prior postnatal depression at which the woman shared her prior experiences of discontinuity and of receiving care from three different health visitors. She explained how discontinuity did not allow her to develop a trusting relationship causing her to refrain from maternity care.

### CMOc 4 –Early initiation

Another generic contextual factor identified in the data was the so-called *“Window of opportunity”* (C), as women were assumed to have a higher degree of mental surplus early in pregnancy than late in pregnancy when focus is on birth-related issues and practical planning. Therefore, the time of initiation was assumed to affect the ability of the intervention to bring about change. Including the women or couples in the intervention early (M resource), i.e., in gestational week 15–18, was hence assumed to evoke the expectant parents’ wish to give their child the best possible start in life, trigger reflections about their coming role as parents and motivate them to make potential changes in life (M reasoning) as outlined by a health visitor:

When you get pregnant and are expecting your first child…, well, then the mental structures are open and that is the most wonderful room for collaboration, because there is also a strong desire to do better than… or to do the best you have learned. There is an opening for collaboration, which is unique in this exact part of life (I_4).

In this excerpt, early intervention (M resource) is described as enhancing the chances for engaging the expectant parents (O). More informants pointed out how addressing the families’ needs early in pregnancy (M resource) was assumed to increase the likelihood of a good initiation of the collaboration and prevent psychosocial risks from developing into circumstances that would be harmful for the children. Positive, motivating experiences at this stage of life (M reasoning) were considered strong determinants of future positive maternal health and wellbeing (O) and thereby of positive parenting and expedient child development (O).

Early initiation of the intervention (M resource) was also assumed to facilitate gentle bridging between sectors as described by this midwife:

I think it makes sense to a lot of pregnant women that we are initiating support in their pregnancies. They may have issues that they worry about in their pregnancy that need to be talked about. That is why it is good to intervene early. It is important for our relationship. Let’s pretend that you have a relationship and share your innermost thoughts with a completely new caregiver right after you have given birth and have arrived home. I think having met before, can make it a little easier for both the mother and the health visitor (I_2).

Meeting the health visitor or other relevant actors before birth was assumed to emphasise feelings of being offered coherent and safe support (M reasoning). Again, these reasonings were described to facilitate trustful relationships between the expectant parents and the HPs and to increase the parents’ chances of benefitting from the intervention (O).

Health visitors and midwives alike described that initiating pregnancy visits by health visitors and performing a coherent intervention relied on well-functioning cross-sectoral collaborations as expressed in the following excerpt:

I think it adds to our job satisfaction knowing each other well. My colleague (a health visitor) takes over for me in the situations that I find difficult. In other situations, I may be the one to provide expert knowledge or suggest a different approach. We support each other through thick and thin (I_19)

Settings where cross-sectoral collaboration was close and integrated through light communication pathways and frequent meetings between HPs (C) were thus identified as positive contexts promoting the implementation of a coherent intervention.

### CMOc 5 –Social networks

Both the existence and quality of networks (C) were experienced by the professionals to affect the expectant parents’ possible intervention outcomes. Some expectant parents in the target group may have been challenged in their ability to form and sustain social relationships, leaving them with a compromised social network. Referring the expectant parents to group courses that typically included attendance of a known HP in informal settings facilitated a social network in safe and non-judgmental surroundings (M resource) as described by a midwife facilitating a baby café for women with psychosocial risks:

The café can be used to facilitate a maternal group because it is a neutral place with no demands. They can come as they are (I_6).

Ultimately, with the accessibility of such a social network, the expectant parents were more likely to experience a sense of commonness and a feeling that they were important to another person (M reasoning). This was expressed by a health visitor in an interview:

Something great about a group course is the development of networks. Suddenly, they make friends […] Many of them are lonely […] They make friends, someone they can talk to, and I know that the health visitor has seen them walk together […] The part about getting to know a few, whom you have something in common with, that is great (I_13).

Experiencing commonness and a sense of being important to another person was considered mechanisms for the expectant parents to benefit from the intervention, as this feeling of commonness was described as generating mirrors, allowing for the exchange of knowledge among parents (O). An informant stated:

The group dynamics are essential as they learn from each other. We see how they learn from each other […] What is going on in these groups add so much more than us visiting the family at home. They are meeting somebody else who more or less face similar challenges being a mum or dad, or who also carries a complicated personal story. Group dynamics are highly essential in this matter (I_14).

Group dynamics were formed by participants with different personalities who had issues fundamentally rooted in the same challenges, which was essential for expectant parents to feel safe and engage in the group sessions. Social networks (M resource) were thus considered as contributing to sustaining the expectant parents’ benefit from the intervention (O).

## Discussion

We applied a realist methodology to identify configurations of contextual factors, mechanisms and outcomes articulated by key stakeholders. Through the development of an initial programme theory, the realist methodology took our findings deep and suggested CMOcs that unearth explanations of how this complex intervention may work, for whom and under which circumstances. The knowledge of how interventions work often remains silent. By eliciting configurations of context, mechanisms and outcomes, this study contributes with the initial step of a realist evaluation that supports the development of an explicit middle range theory base for early interventions for expectant parents with psychosocial risks. The initial programme theory is left to be tested and refined in different contexts with attention being given to both intended and unintended outcomes as well as harmful consequences thus contributing to the safety of such interventions.

CMOc 1 suggests that acknowledgement, use of non-judgmental communication and a respectful, sensitive approach by HPs are generative mechanisms for the intervention to produce effect. This finding is in line with other recent studies that show such a care approach is key to ensuring that expectant parents with psychosocial risks engage with antenatal services [18, 23, 59] as experiences of being judged or labelled by HPs are frequent among this group [15, 17]. Being offered participation in a supportive intervention may be a stigmatising experience for expectant parents with psychosocial risk factors since the professional risk-assessment may not be in line with their own assessment and may clash with their self-image [17, 60]. The intervention may also negatively affect their self-belief and perception of parental skills [15, 23]. As reported by Mule *et al*. [18] and Frederiksen et al. [17], fear of being judged or perceived as bad parents may lead expectant parents to not share important information with professionals or to avoid disclosing problems they are experiencing. These studies underpin why HPs emphasise the value of keeping a respectful and non-judgmental approach when caring for expectant parents. They also help identify a potentially unintended consequence of the intervention, i.e., that expectant parents identified with psychosocial risk factors may feel stigmatised or labelled into a category they do not recognise.

CMOc 2 shows that clear and well-timed communication may contribute to a feeling of being in control among expectant parents with psychosocial risks. Accordingly, Rayment-Jones et al. found that women with complex social risk factors may perceive maternity services as a system of surveillance rather than as support and that building relationships with HPs may be intimidating and appear as a loss of control in their lives [59]. Freedom of choice in antenatal interventions is questioned by some authors [61]. Specifically, these authors found that offers of support are not always experienced as optional [61] due to the power relation that evolves within all social relations [62]. This reflection emphasises that expectant parents with psychosocial risks may attend supportive services, not simply from freewill but due to a fear of consequences if they decline to participate. Another potentially unintended consequence of the intervention may be that expectant parents may feel forced to attend the intervention regardless of their personal interests. Their concern and distress may furthermore be exacerbated by worrying about possible implications related to sharing of sensitive information or declining participation in supportive services.

Through CMOc 3, continuity of caregiver was described as an important generative mechanism for expectant parents feeling safe and developing trustful relationships with their care providers. With continuity of HP as a central ingredient, the assumption underlying this CMOc gains support from studies which show how antenatal care programmes based on continuity of care provider appear to be successful in addressing complex care needs of the women with psychosocial risk factors thereby improving birth outcomes such as gestation and birth weight [19, 63, 64]. An ongoing relationship between a patient and a care provider, defined as relational continuity [65], does not constitute continuity or ensure care coherence alone. Other important aspects relate to transfer of information (information continuity) and the overall organisation of care (management continuity) [65]. This is especially important when care is received from various providers [65], as seen in the intervention discussed here, and management continuity therefore becomes extraordinarily important. This finding was confirmed in a recent study of continuity of care for expectant parents receiving the present intervention [66]. It is thus likely that a systematic approach to cross-sectoral collaboration combined with well-established collaboration between the involved HPs may bring about a coherent and meaningful antenatal care pathway. This perspective emphasises a key intention of the intervention: Bridging the gaps between the sectors and fostering a gentle transition from midwife (regional service) to health visitors (municipal service) and other relevant municipal or regional actors to whom the pregnant woman may be referred.

CMOc 4 is closely linked with CMOc 3 as early initiation leaves time for early introduction to relevant care providers, the identification of needs and initiation of support, collaboration and continuity across sectors, and services when parents are open to change. In accordance with research literature [65, 67], early initiation also supports the building of trust as an outcome of continuity because it leaves time for familiarity between parents and caregivers. Continuity of care models have been identified as important in the contexts of detecting perinatal mental health problems, leaving room for establishing a trustful relationship and giving HPs the possibility to draw on previous encounters with the family [19, 67]. Continuity of care models are, however, time demanding. Considering that midwives and nurses in perinatal care settings indicate limited time as the most dominant barrier to addressing mental health issues with pregnant women [68], attention should be given to potential challenges such as time restrictions when aiming to provide continuity in the care for the expectant parents in the intervention undergoing this evaluation.

CMOc 5 suggests that available social networks may lead to better learning opportunities for parents because of both the chances for mirroring and exchanging of knowledge and the positive and supportive aspect that a feeling of belonging provides. This hypothesis is further emphasised by Berkman et al. [69] who elaborate on social networks as an opportunity for social support which has been shown to impact health behaviour. A key element in Berkman et al.’s theory is the distinction between supportive and non-supportive social relations [69] as exemplified through research literature showing that perceived social support is a significant protective factor for maternal mental health including post partum depression [70]. Thus, research literature supports our findings by emphasising the positive impact of social support among new parents. Our findings suggest that relational difficulties exist particularly among expectant parents with psychosocial risks. Components of the intervention acting to increase accessibility of social networks are therefore assumed to provide opportunity for expectant parents to reach out to other likeminded parents to fulfil their need for support and belonging. For whom, how, why and under what circumstances these opportunities may work will be tested in the evaluation, which is ongoing.

HPs possessing specialised knowledge and communicative skills are suggested as an important moderating contextual factor in the initial programme theory. This assumption is supported by Turienzo et al. [64] who found that organisational infrastructure, such as training and support of the staff and access to guidelines, was essential to ensuring high quality of care and for addressing the complex care needs of pregnant women with either low socioeconomic status or social risk factors. In building the programme theory, front-line midwives repeatedly highlighted the importance of training and experience affecting their delivery of the intervention with confidence. The importance of this contextual factor is underpinned by international studies reporting that midwives do not feel equipped to provide mental healthcare or perform psychosocial risk assessments [67, 68, 71]. The same studies also provide support for the assumption that having enough time to build trust and conduct quality psychosocial risk assessment is important for ensuring that expectant parents experience the intended intervention outcomes. Additionally, studies indicate that limited ability to perform quality maternity care may be a source of stress, reduce job satisfaction and ultimately cause job resignation [72, 73] among HPs. Such potentially unintended and harmful consequences warrant further attention in the theory-testing phase of this evaluation.

From a socioecological perspective [74], the intervention mainly works at an individual level and targets expectant parents with psychosocial risks. The initial programme theory, however, indicated pathways reaching beyond the expectant parents’ engagement with the intervention, including organisational level components such as improved professional competencies and cross-sectoral collaboration and the accessibility and quality of a supportive social network. Early indications suggest that there may be differences in parents’ experiences depending on their psychosocial risks and life experiences influenced by structural, organisational and individual contextual factors.

The intervention positions itself as a high-risk prevention strategy [75] as it aims to improve the maternity care for expectant parents who are at a high risk of experiencing adverse birth outcomes and negative maternal and child health outcomes. The intervention strives to reduce this risk by improving the woman and her partner’s ability to attach to their child. This is achieved by supporting and improving their coping ability and parental skills via extended and/or shared consultation, health visits and individual- and group based tailored services. *“The priority of concern should always be the discovery and control of the causes of disease”* [71: p. 432]. By taking into account that health is widely influenced by distal, societal structures, the effect and—more importantly—the sustainability of an intervention may be supported by focusing on eliminating fundamental causes of disease rather than proximal risk factors [76, 77]. According to the initial programme theory, the intervention is identified with only limited focus on more fundamental causes of disease. Following both a complex system [78] and a socioecological perspective [74], a risk exists that the intervention was designed to focus on casual pathways that do not hold the power to provide sustainable, long-term outcomes. Another limitation of high-risk prevention strategies is their dependency on the individual’s contribution thus leaving the intervention user with the responsibility for benefitting from the intervention [75]. In this respect, the intervention may cause an effect opposite to what was intended and increase social inequality in maternity, as expectant parents with psychosocial risk factors may not have the resources to meet the demands embedded in the intervention. Adding a focus on underlying and fundamental risk factors via collaboration with relevant services across sectors and supporting parents’ access to e.g., financial support [79], education or labour market may increase the likelihood that the intervention reaches its long-term overall objective of reducing social inequity of health. Attention to the reach of the intervention in relation to the parents’ risk factors will be a focus of the ongoing realist evaluation. We identified the five most dominant generative mechanisms assumed to be essential for the intervention to produce the intended outcomes. While considering potentially unintended consequences identified through this study, the CMOcs constitute the framework for the ongoing evaluation [37].

A remarkable strength of this study is that the theories generated in this first phase of the realist evaluation were based on multiple data collection methods and sources of evidence. In qualitative studies, validity may be addressed through the extent of triangulation. Triangulation of the data collection methods was thus expected to promote rich data with an appropriate scope while capturing the complexity that resembled the intervention and contributed to a strong theoretical base [80].

Another strength of this study is the use of multiple stakeholders’ involvement in prioritisation of the elements of the programme theory. It may be difficult to determine how changes are brought about because multiple stakeholders are involved, and their responses are often not aligned [81]. To reduce the risk of identifying and prioritising incorrectly, we included key stakeholders in workshops thus facilitating the processes of choosing the aspects that were considered pivotal in the initial programme theory. The workshops helped us gain a better understanding of the interdependencies of the mechanisms and allowed us to grasp how they were often embedded within one another. Stakeholder involvement leads to mutual understandings and influence [82]. The workshops eventually allowed the stakeholders to reflect on and gain insight into the entire intervention beyond their own setting.

A potential limitation of the study relates to the fact that although we obtained assumptions from programme designers and stakeholders representing the decisive level and front line workers, we did not include that of expectant parents. Parents from all participating municipalities will be included as stakeholders in the theory-testing phase of the evaluation. [29, 54].

## Conclusion

This study constitutes the starting point of an evaluation of an intervention for expectant parents with psychosocial risk factors by theorising how, for whom and under which circumstances it is expected to work. The intervention is expected to reduce adverse birth outcomes and support positive child development. Through the initial programme theory, we identified the key assumption that trust building is essential for expectant parents living with psychosocial risks. Trust building allows expectant parents to feel sufficiently safe to engage with the services thereby improving their parental skills and ensuring that they feel less stressed. Qualifications of the HPs, such as their communicative skills, were identified as contextual factors expected to stimulate an acknowledging, respectful and non-judgemental approach which provided room for the HPs to establish a trustful relationship with the expectant parents. A generative contextual condition seemed to be HPs’ ability to establish interprofessional and cross-sectoral collaboration as a well-functioning collaboration across professions which was essential in regard to early initiation, gentle bridging and continuity of care. Finally, the expectant parents’ past experiences with the healthcare system were considered as an influential context for how they respond to the intervention. This relates to the acknowledging, respectful and non-judgemental approach of the HPs involving timely communication. Potential unintended consequences that warrant further attendance in the theory-testing phase of the evaluation include the parents’ experiences of stigma, marginalisation or loss of control. Such experiences may potentially threaten the intended intervention outcomes and ultimately increase health inequality for both parents and children.
